# *RSC Advances*: celebrating 10 years of publication

**DOI:** 10.1039/d1ra90127c

**Published:** 2021-07-09

## Abstract

Celebrating the 10th anniversary of RSC Advances.
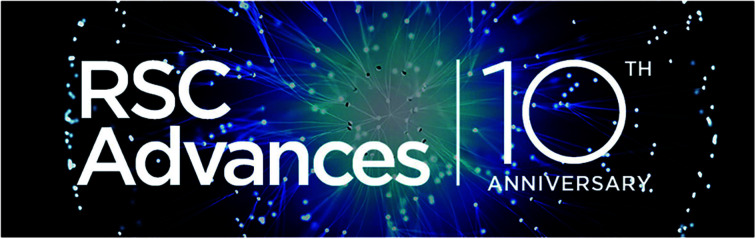

In July 2011 the Royal Society of Chemistry published the first issue of *RSC Advances*,^1^ so this month marks our 10^th^ anniversary! Since 2011 we’ve published over 62 000 articles across the breadth of the chemical sciences. We’ve got a lot of activities planned to celebrate our 10^th^ year, but we’d like to start by sharing some of the history of our journal, and how we got to where we are today.

## Humble beginnings

*RSC Advances* was launched in 2011 to expand the RSC’s reach in the chemical sciences, providing a high-quality publishing option for all sections of our community, including emerging scientific areas and markets. Research on chemistry topics outside of the scopes of the existing RSC journal portfolio was actively encouraged, and there were no requirements around whether work should be of interest to a broad, or more specific audience. We wanted to cover everything in *RSC Advances*, and attract as wide a readership as possible. The journal supports early-career researchers and researchers from nations that are developing their research base. As now, the main criteria are that the work is well-conducted, contains chemistry or is of relevance to chemists, and advances the development of its field.

From the start we always tried to be innovative. The journal was launched with behind-the-scenes topic modelling software, meaning all articles are classified by their subject area. This makes it easier for readers to find content relevant to them, and to sign up to subject-specific table of contents alerts. In 2013 we moved to article-based publishing, such that all articles are assigned an issue and page numbers immediately upon publication, with no waiting around for the final paginated version.

## Rapid growth

**Fig. 1:**
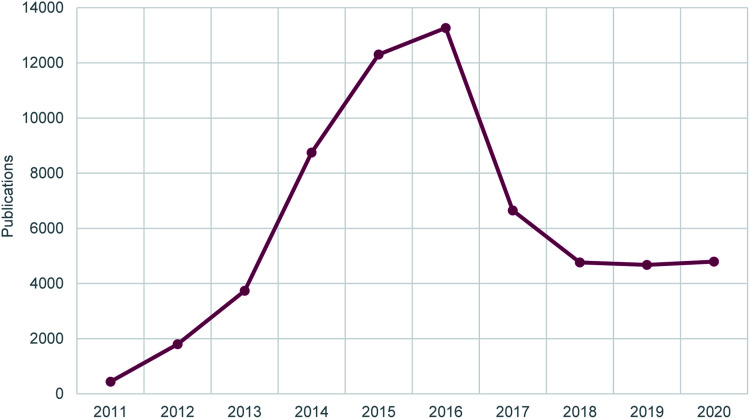
Number of publications in *RSC Advances* since launch in 2011.

*RSC Advances* grew rapidly in its first few years, and between 2013 and 2016 this growth became even steeper (Fig. 1). The journal was becoming more and more popular with new authors, and also accepted transfers from other RSC journals, offering authors an easy alternative route to publish research with the RSC in cases where that research was deemed good quality but less urgent, or out of scope for the journal initially submitted to. *RSC Advances* quickly became the world’s largest chemistry journal.††Based on 2016 output of all journals categorised in a core chemistry category in Web of Science.

In order to keep up with the increasing numbers of submissions to *RSC Advances*, a change in editorial structure was needed. In 2015 the journal converted from a professional editor model to an Associate Editor model, and we hired around 200 Associate Editors to work with us on the journal. We also launched a reviewer panel^2^ – a group of dedicated reviewers with expertise across the journal’s scope, who help support our Associate Editors in carrying out peer review.

## Becoming an open access journal

The decision to make *RSC Advances* gold open access (OA) involved a number of interconnected considerations.^3^ The way scientists communicate their research has naturally evolved in the last ten years, with the demand for open access publishing on the increase.^4^ Many funding bodies and institutions also now require authors to publish their research under an open access model.^3^ As a learned society publisher, it is important that we provide our community with OA journals to fulfil their needs and requirements. By 2017 *RSC Advances* was a trusted journal with well-established and mature editorial practices, and we believed this would greatly enhance the chances of a successful conversion to open access. In addition, although we do not view *RSC Advances* as a mega-journal, because it does not operate an ‘objective’ peer-review process, it is of a comparable size and scope to well-established and trusted mega-journals. The journal’s size and scope gave us confidence that 2017 would be the right time to make the transition.

To further support the transition to a more open research environment and as a benefit to our community, we set one of the lowest article processing charges (APCs) in the industry, as well as offering support to authors from lower income countries through discounts and waivers.

By converting *RSC Advances* to a gold OA journal, we were able to evolve alongside the demand, and disseminate quality research to the largest possible audience, maximising its visibility. The transition has also allowed us to help shape the future of open access publishing and support our community in the transition to OA.

## Maintaining consistency

Despite the vast changes *RSC Advances* has gone through in its short history, one thing has always remained the same: our commitment to publishing quality research across the breadth of the chemical sciences, and to making our journal as accessible as possible to our authors and readers.

**Fig. 2:**
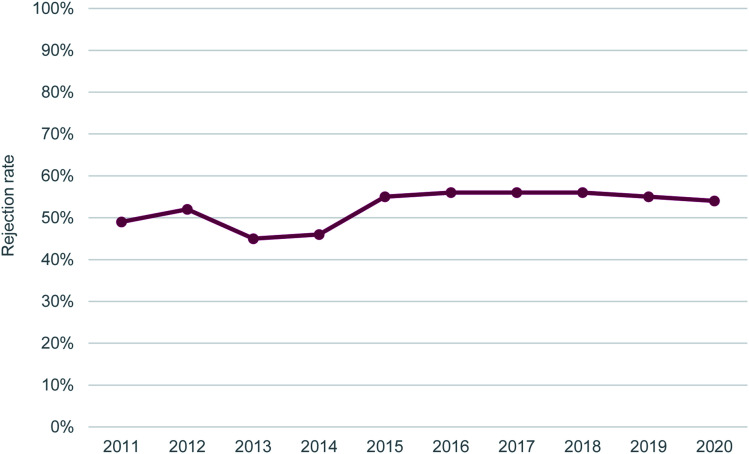
*RSC Advances*’ rejection rate between 2011 and 2020.

**Fig. 3:**
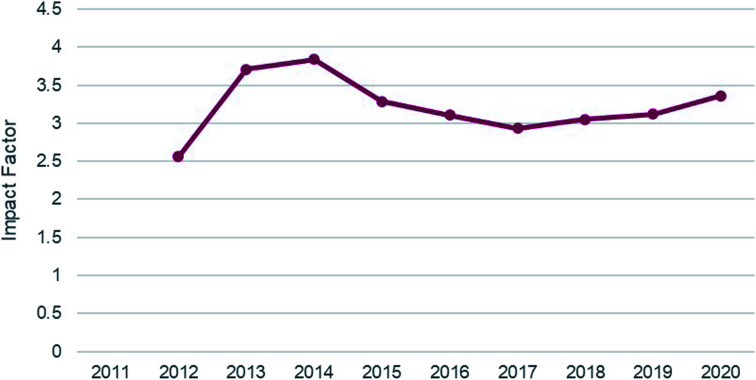
*RSC Advances*’ impact factor between 2011 and 2020.

The journal’s rejection rate has remained consistent at ∼55% since 2015 (Fig. 2), and our Impact Factor has stayed between 2.9 and 3.2 in the same time period (Fig. 3). Our scope hasn’t changed since we launched in 2011 – we still publish quality work across the breadth of the chemical sciences.

We believe this consistency demonstrates our commitment to maintaining high standards, and to providing a great home for research across the chemical sciences. It shows that open access publishing is just as reliable as the subscription model, and that we continue to offer a great service to our authors and readers.

## A society publisher

As well as being a trusted journal with a commitment to quality publishing, there are many other benefits^5^ to publishing with *RSC Advances*, and with the Royal Society of Chemistry in general. We are a society publisher, meaning that as well as our journals, we also provide other services and resources for members of the chemistry community, from grants and awards, to tailored support for chemistry teachers and people working industry. Key initiatives launched in recent years include the RSC’s bullying and harassment support service^6^ and our joint commitment for action on inclusion and diversity in publishing.^7^

Corresponding authors who pay an APC to publish in *RSC Advances* are offered a year’s free affiliate membership of the Royal Society of Chemistry, so they can take advantage of some of the benefits we offer, such as membership of subject interest groups, career advice, and mentoring.

## The future

The future looks bright for *RSC Advances*. As well as maintaining the consistency that has allowed us to be successful over the past few years, there are areas in which we think we can still improve.

Inclusion and diversity is a key focus for the Royal Society of Chemistry, and we see *RSC Advances* as a big part of that. Though we are proud of some aspects of our diversity, such as the 50 : 50 gender balance on our Editorial Board, and the fact that we published papers from across 172 different countries in 2020, we know that we can still do more, and we hope to take some positive steps in the upcoming months.

We also want to improve our engagement with the younger generation of scientists. They are the future of our field, and we want to celebrate them and their research. We’ve recently launched our Emerging Investigators series,^8^ which we hope will be a great success, and we’ve had some fantastic young chemists writing for our blog.^9^ Our reviewer panel is another opportunity for early career chemists to be involved. We are looking to expand that in 2021, and welcome new applications.

We hope you’ve enjoyed reading this recap of *RSC Advances*’ 10 years of publication, and that you will enjoy reading the articles that follow. We wouldn’t be where we are today without the support of our authors, readers, and reviewers, so we want to offer our thanks to every one of you. Here’s to the next 10 years!

Dr Laura Fisher, Executive Editor, *RSC Advances*

Professor Russell Cox, Editor-in-Chief, *RSC Advances*

## Supplementary Material
